# Effect of Low-Dose Alcohol Consumption on Chronic Liver Disease

**DOI:** 10.3390/nu16050613

**Published:** 2024-02-23

**Authors:** Silvia Andaloro, Fabrizio Mancuso, Luca Miele, Giovanni Addolorato, Antonio Gasbarrini, Francesca Romana Ponziani

**Affiliations:** 1 Liver Unit, CEMAD Centro Malattie dell’Apparato Digerente, Medicina Interna e Gastroenterologia, Catholic University, Fondazione Policlinico Universitario A. Gemelli IRCCS, 00168 Rome, Italy; silvia.andaloro01@icatt.it (S.A.); fabrizio.mancuso01@icatt.it (F.M.); francescaromana.ponziani@policlinicogemelli.it (F.R.P.); 2 Department of Abdominal, Endocrine and Metabolic Medical and Surgical Sciences, Fondazione Policlinico Universitario A. Gemelli IRCCS, 00168 Rome, Italy; luca.miele@policlinicogemelli.it; 3 Department of Translational Medicine and Surgery, Catholic University, Fondazione Policlinico Universitario A. Gemelli IRCCS, 00168 Rome, Italy; giovanni.addolorato@policlinicogemelli.it; 4 CEMAD Unit, Digestive Disease Center, Fondazione Policlinico Universitario A. Gemelli IRCCS, 00168 Rome, Italy; 5 Internal Medicine and Liver Transplant Unit, Fondazione Policlinico Universitario A. Gemelli IRCCS, 00168 Rome, Italy; 6 Internal Medicine and Alcohol Related Disease Unit, Columbus-Gemelli Hospital, Fondazione Policlinico Universitario A. Gemelli IRCCS, 00168 Rome, Italy

**Keywords:** alcohol consumption, chronic liver disease, NAFLD, viral hepatitis, HBV, HCV

## Abstract

Although alcohol is one of the most important etiologic agents in the development of chronic liver disease worldwide, also recognized as a promoter of carcinogenesis, several studies have shown a beneficial effect of moderate consumption in terms of reduced cardiovascular morbidity and mortality. Whether this benefit is also present in patients with liver disease due to other causes (viral, metabolic, and others) is still debated. Although there is no clear evidence emerging from guidelines and scientific literature, total abstention from drinking is usually prescribed in clinical practice. In this review, we highlight the results of the most recent evidence on this controversial topic, in order to understand the effect of mild alcohol use in this category of individuals. The quantification of alcohol intake, the composition of the tested populations, and the discrepancy between different works in relation to the outcomes represent important limitations emerging from the scientific literature. In patients with NAFLD, a beneficial effect is demonstrated only in a few works. Even if there is limited evidence in patients affected by chronic viral hepatitis, a clear deleterious effect of drinking in determining disease progression in a dose-dependent manner emerges. Poor data are available about more uncommon pathologies such as hemochromatosis. Overall, based on available data, it is not possible to establish a safe threshold for alcohol intake in patients with liver disease.

## 1. Introduction

Excessive alcohol use is a known deleterious factor for human health, causing a considerable number of deaths each year [1,2,3]. It is also recognized as an important risk factor for carcinogenesis, particularly for oropharyngeal, laryngeal, and gastrointestinal neoplasms, but with a detrimental effect observed in several other types of cancer [4,5,6,7]. A debated topic among experts is whether a low dose of alcohol consumption (e.g., intake below the threshold that allows us to make a diagnosis of alcoholic liver disease according to the European Association for the Study of the Liver [EASL] guidelines [8]) may be safe and even beneficial in some cases. Many researchers have conducted studies to answer this question, leading to heterogeneous results. A contrast persists between those who argue that even modest intake may be a determinant in the occurrence of adverse outcomes and those who believe that a positive effect is evident, especially on the basis of several studies that have shown a reduced incidence of major cardiovascular (CV) events and lower mortality from CV causes, although no long-term randomized trial has been conducted in this context [9,10,11,12,13].

The issue becomes even more debated when it is extended to individuals with liver disease in whom another pathogenic cause (viral, metabolic, or other) is already present. Since the diagnosis of alcohol-related liver disease is based on evidence of clinical and biochemical abnormalities in association with alcohol consumption exceeding certain limits (set at thirty grams per day for males and twenty grams per day for females), it should be evaluated if the assumption of drinks in a quantity below these thresholds could have detrimental consequences in this category of patients.

In this review, we aim to analyze the evidence about the effects of low-dose alcohol consumption in patients with a pre-existing liver disease.

## 2. Drinking Quantification

It is very problematic to make an accurate assessment of the amount of drinks consumed by an individual. This is mainly due to a major self-report bias, partly caused by the difficulty of obtaining true information on the grams of alcohol contained in different types of drinks, with a coexisting lack of reliability in reporting the amount of each of the various types of drinks taken by patients. To overcome this limitation, it is reasonable to avoid estimating exact grams while it is more useful to quantify the number of standard drinks taken. In the many national and international guidelines, the definition of alcoholic unit (or standard drink) is not in agreement, with values ranging from eight to sixteen grams. The most accepted measure is that proposed by the World Health Organization (WHO), which has established this amount as ten grams of pure alcohol, corresponding approximately to a half glass of wine (125 milliliters with an alcohol content of 12°), a can of beer (330 milliliters at 4.5°) or a glass of hard liquor (40 milliliters at 36°) [14].

Drinking habits can be assessed through interviews, quantity frequency diaries, or questionnaires. A viable alternative to these methods is the use of screening instruments routinely employed to identify alcohol use disorder. The most widely adopted instrument is the Alcohol Use Disorders Inventory Test (AUDIT), which consists of ten questions, the first of which is the Alcohol Use Assessment Test [15].

## 3. NAFLD

Non-alcoholic fatty liver disease (NAFLD) is a widespread condition, with a worldwide estimated prevalence ranging from 6 to 35 percent. NAFLD is nowadays the most common cause of liver disease in Western industrialized countries, due to the broad diffusion of metabolic risk factors like diabetes mellitus, arterial hypertension, dyslipidemia, and obesity [16,17,18,19,20]. The spectrum of NAFLD varies from simple hepatic steatosis to non-alcoholic steatohepatitis (NASH), in which there is a coexistence of fat accumulation, lobular inflammation, ballooning of the hepatocytes, and fibrosis, to liver cirrhosis [21,22,23].

Even if there are no guidelines or evidence in favor of or against the consumption of a modest amount of alcohol in these patients, in clinical practice, a total abstention from drinking is often recommended.

It should be noted that several studies indicated cardiovascular disease as one of the main causes of mortality in patients with NAFLD [24] and that the use of a moderate quantity of alcoholic beverages has a beneficial effect in decreasing unfavorable cardiovascular outcomes in the general population [25]. Following these considerations, the discussion about this controversial issue is as current as ever, and many observational studies have been carried out. The main characteristics of the analyzed studies are summarized in Table 1.

Sookoian et al. performed a meta-analysis including 43,175 individuals, which showed a protective effect of minimal alcohol consumption against NAFLD, more evident in women than in men, and against the development of advanced disease [26].

**Table 1:** Studies on NAFLD patients.

Reference	Population Sample	Modest Alcohol Use Definition	Main Results
Chang et al. 2019 [27]	58,927	<30 g/d in men <20 g/d in women	progression of fibrosis
Kawamura et al. 2016 [28]	9959	<40 g/d	increased risk of HCC development in modest alcohol users
Moriya et al. 2015 [29]	5297	<280 g/w	protective effect of alcohol use against steatosis in men (in women only if <70 g/w)
Åberg et al. 2019 [30]	8345	<30 g/d in men <20 g/d in women	increased liver events if intake is above 9 g/d; reduced CV mortality in drinkers (up to 49 g/d)
Hajifathalian et al. 2019 [31]	4568	<1.5 drink/d	reduced risk of death in moderate consumers
Younossi et al. 2019 [32]	4264	>3 drinks/w <2 drinks/d for men	only excessive alcohol consumption increases death rate
Akahane et al. 2020 [33]	2429	<60 g/d	alcohol use is inversely associated with NAFLD
Patel et al. 2017 [34]	151	<20 g/d	no protective effect of moderate alcohol use

A large cohort study conducted in Korea, involving more than 58,000 individuals with fatty liver disease in abdominal ultrasounds and an estimated low probability of fibrosis according to non-invasive markers (FIB-4, NFS, and APRI) [27], demonstrated a correlation between light (1–10 g/day) or moderate alcohol consumption (10–30 g/day in males, 10–20 g/day in females) and the progression to intermediate or high fibrosis. When the latter of these two outcomes was used as the unique endpoint, the aforementioned association was even stronger. To exclude the possible effect of binge drinking, a subgroup analysis on patients who did not consume 60 or more g of alcohol on a single occasion was performed, confirming an increased risk of fibrosis progression in moderate drinkers. An important limitation of this work was the composition of the sample, predominantly consisting of young middle-aged individuals, which makes it difficult to generalize the results to the overall population.

Another large cohort, including nearly ten thousand patients with an echographic diagnosis of steatosis, was analyzed in Japan. The aim was to evaluate the effect of drinking on the incidence of hepatocellular carcinoma (HCC) [28]. The population was stratified according to the daily amount of alcohol intake, and a higher incidence of HCC at increasing levels of ethanol consumption was observed. In the multivariate analysis, drinking 40 or more g/day of alcohol was reported as an independent risk factor for carcinogenesis. These results have to be cautiously interpreted because of the imbalance between sex categories in the cohort, with a large prevalence of men; another limit is the lack of a comparison with the incidence of HCC in the general population.

A similar study included 5297 subjects who underwent an ultrasound examination of the liver during a systemic health check-up conducted in Japan [29]. Participants were categorized according to the weekly quantity of ethanol assumed and divided into four groups (0–1–69.9, 70–139.9, 140–279.9, and more than 280 g/week). The results showed an inverse relationship between each category and fatty liver in males, whereas in women, only an intake below the threshold of 70 g per week appeared to be protective against steatosis. Drinking frequency was also evaluated: in men, an inverse association with NAFLD irrespective of the number of days of alcohol consumption was seen; drinking on 4 to 6 days per week and on a daily basis inversely correlated with fatty liver in women. The main limitation of this work is the composition of the sample, which consisted of healthy individuals, thereby making these results impossible to apply to the general population.

Aberg et al. analyzed a cohort of more than eight thousand individuals with NAFLD, using the fatty liver index (FLI) as a diagnostic criterion. The endpoints were fatal and non-fatal liver-related events (requiring hospital admission or causing liver cancer or death), cardiovascular events, cancer, and all-cause death [30]. An alcohol intake above 9 g per day had a statistically significant association with adverse liver outcomes. An increased risk for the onset of cancer was observed in people consuming more than 30 g of ethanol per day. An alcohol intake of up to 49 g/day was associated with a reduced risk for incident cardiovascular adverse events, and a J-shaped correlation with all-cause death was found, with a maximal risk reduction of 21% in the low-consumption category (from 0 to 9 g per day); reaching an intake above 30 g/day, the risk of death tended to increase proportionally to the rising quantity of alcohol consumed. In the subgroup of patients who consumed less than 24 g per day of ethanol, the largest risk reduction for cardiovascular death and mortality of all causes was seen in preferential wine drinkers. In addition, whereas consumption of non-wine alcoholic beverages up to 24 g/day raised the risk for liver events, low preferential wine drinking exhibited nonsignificant risk estimates for liver disease.

A study on a cohort obtained from the National Health and Nutrition Examination Survey (NHANES, a nationwide survey of the American population) was performed. The sample consisted of 4568 individuals affected by NAFLD (diagnosed with HSI score) [31]. Modest alcohol consumption, defined as an intake of a minimum of half a drink to less than one and a half drinks per day, was associated with a significantly lower risk of death; conversely, drinking a higher amount of alcohol showed a trend toward increased mortality. A separate evaluation according to the sex category was carried out, with evidence of a more relevant beneficial effect of modest alcohol consumption in women rather than in men. Among older patients (people above 64 years), drinking more than a modest quantity of ethanol did not show a harmful effect on mortality. The population was also stratified according to FIB-4 score values: in subjects at high risk of fibrosis, no beneficial effect of modest drinking was observed. Even excluding deaths from cardiovascular causes, the aforementioned protective effect remained; similarly, the deleterious effect of drinking more than one and a half drinks per day was confirmed.

Another study was based on the NHANES database, comprising more than 4000 individuals with evidence of steatosis in abdominal ultrasounds [32]. Only excessive alcohol consumption (higher than 3 drinks/day for men) was independently associated with an increased overall mortality. Analyzing the specific causes of death, only mortality by cancer was found to be correlated with excessive alcohol intake; in patients affected by metabolic syndrome, there was also an association with fatal cardiovascular outcomes. Regarding the consumption pattern, it was seen that binge drinking (defined as having 5 or more alcoholic drinks on a single occasion) for at least 13 days a year implied a significant rise in the risk of death, which becomes even higher in the case of binge drinking for more than 20 days per year.

Akahane et al. analyzed a cohort of 2429 males with an ultrasound diagnosis of fatty liver during an annual health check-up [33]. The prevalence of NAFLD was shown to be the lowest in the moderate-drinking group (alcohol intake of 30–60 g/day) and the highest in the abstainers. Chronic alcohol consumption was independently and inversely associated with liver steatosis, with a greater beneficial effect seen in light drinkers (defined as people who consumed less than 30 g/day of alcohol). The absence of women also makes it difficult to generalize these results in this case.

A small study including 151 diabetic patients with NAFLD (diagnosed according to the presence of steatosis detected with ultrasound), with a non-invasive evaluation of liver stiffness made by transient elastography, reported no significant protective effect of light or moderate alcohol consumption [34].

### 3.1. Histological-Based Studies

All the aforementioned studies share the characteristic of a diagnosis obtained with non-invasive methods. However, other studies on smaller populations of patients with a biopsy-proven diagnosis of NAFLD were carried out. Table 2 highlights the main features of these works.

In particular, abstinent participants of trials conducted by the “Nonalcoholic Steatohepatitis Clinical Research Network” (NASH-CRN) undergoing at least two liver biopsies separated by at least 12 months had a higher probability of amelioration of aspartate transaminase (AST) levels and histologic steatosis grade compared to modest drinkers. Interestingly, more than monthly alcohol users had a greater improvement in portal inflammation in comparison to those who drank monthly or less frequently. It was demonstrated that among patients affected by NASH at baseline, non-drinkers were most likely to obtain a histologic resolution, whereas modest drinkers had lower rates of recovery. Additional important data were observed in previous alcohol users who reported abstention at follow-up, with the evidence of a resolution percentage not so far from that of lifetime abstainers [35].

Another cross-sectional study performed using the database of NASH-CRN, including 583 subjects with a biopsy-proven diagnosis of NAFLD [36], reported a protective effect of modest alcohol use on the onset of steatohepatitis; this association became stronger in those who drank more frequently during the week. The beneficial effect was present both in males and females.

An analysis of a cohort of about three hundred Japanese patients, categorized as abstainers and mild drinkers (with a consumption below the threshold of 20 g/day) showed a higher prevalence of histologically assessed cirrhosis in the second group [37]. The incidence rate of hepatocellular carcinoma (HCC) was higher among mild alcohol users, and it was concluded that drinking even a small amount of alcohol is an independent risk factor for hepatocarcinogenesis.

Similar results were reported by the analysis made by Ascha et al. on a group of almost two hundred individuals referred for liver transplant because of NASH cirrhosis [38]. In fact, it was demonstrated that any lifetime alcohol consumption was an independent risk factor for the development of HCC, with 3–4 times increased odds in comparison to abstainers.

Other data were derived from severely obese patients who underwent liver biopsy during preoperative assessments for bariatric surgery. Light-to-moderate alcohol consumption (up to a maximum of 200 g/week) in these subjects seemed to reduce the probability of histologically demonstrated NASH [39]. A cross-sectional study with a similar design including more than one hundred patients affected by morbid obesity showed no significant correlation between the entity of alcohol intake and histopathological findings, thereby excluding a beneficial effect of drinking [40].

Another recent analysis including 187 individuals with a biopsy-established diagnosis of NAFLD [41] revealed that, compared to lifetime abstainers, patients categorized as modest (defined as consuming less than 70 g/week of alcohol) or moderate (70–140 g/week of alcohol for women, and up to 210 g/week for men) had a lower prevalence of advanced fibrosis. In multivariate analysis, only modest alcohol use was recognized as a protective factor. Regarding the drinking pattern, this beneficial effect was observed only in non-binge drinkers, namely those who introduced less than four or five standard drinks (for females and males, respectively) on a single occasion. Considering the type of alcoholic beverage, a protective effect against liver fibrosis was proven in exclusive wine consumers but not in beer drinkers.

A small longitudinal study comprising 71 individuals with histologic evidence of steatosis who repeated a second liver biopsy at a follow-up [42] also demonstrated that only heavy episodic drinking (more than 60 g of alcohol for men and 48 g for women in a single day) was proven to be an independent risk factor for fibrosis progression.

In another cohort with a similar number of biopsy-proven cases of NAFLD [43], the consumption of less than 24 g/year of alcohol was an independent risk factor for severe liver disease, whereas patients who drank a higher amount had a lower risk of fibrosis and cirrhosis. Even an intake above the lower threshold of 10 g/year guaranteed this beneficial effect, which was lost in drinkers of more than 40 g/year.

### 3.2. Analysis of Surrogate Markers of Alcohol Consumption

An important bias that could affect all the aforementioned studies is the evaluation of the amount of alcohol consumption, obtained with the administration of questionnaires. To overcome this considerable limitation, some researchers measured the blood levels of phosphatidyl ethanol (PEth), an alcohol-related marker.

Based on this method, in a cohort of 139 patients who underwent liver biopsy as part of a NAFLD severity workup [44], an intake limit of up to 13 drinks per week was associated with a decreased probability of advanced-stage fibrosis for each adjunctive alcohol unit. The study population was divided into quartiles according to the entity of ethanol consumption, with the second one represented by those who drank more than 0.3 but less than 1 alcohol unit per week. Taking this quartile as a reference, the protective effect previously described was present in the highest quartiles but not in the lowest. The thirteen subjects with PEth values above the cutoff value of 0.3 μmol/L had an increased risk of disease progression (in terms of fibrosis) in comparison to the other subjects. 

The same biomarker was dosed in another study conducted on a sample of 86 histologically ascertained cases of steatosis [45]. The results showed that moderate alcohol consumption, regardless of whether it was assessed with questionnaires or with the measurement of PEth, with a threshold of 50 ng/mL, led to an increased risk of advanced fibrosis.

An interesting analysis was carried out on a cohort of 266 patients with histopathological evidence of NAFLD to evaluate the polymorphism rs1229984 G > A (G being the ancestral allele) in the gene that encodes for the alcohol dehydrogenase ADH1B, which has been robustly associated with the level of alcohol consumption [46]. Carriers of the A (His-48) allele are at low risk of alcohol dependence and consume low levels of alcohol because the variant is associated with increased activity of the enzyme, leading to more rapid oxidation of ethanol to acetaldehyde, making them more likely to find drinking unpleasant. It was observed that carriers of this allele had a significantly lower degree of histological steatosis and scores of necroinflammatory activity when compared with noncarriers, in the absence of a correlation with fibrosis stage. In conclusion, data showed no beneficial effect of genetically predicted light-to-moderate alcohol consumption on the histological outcomes in these subjects.

### 3.3. Biochemical Pathways Triggered by Alcohol in NAFLD

Other studies were developed to elucidate some mechanisms that could underlie the possible beneficial consequences of minimal alcohol consumption. In patients with biopsy-confirmed NAFLD [47], light alcohol consumers (defined as drinkers of less than 20 g/day of alcohol) had a lower prevalence of NASH compared to abstainers. Furthermore, a microarray analysis of randomly selected individuals found an important inhibition of pathways involved in the immune response and an associated reduced activity of monocytes in the light alcohol group.

**Figure 1:**
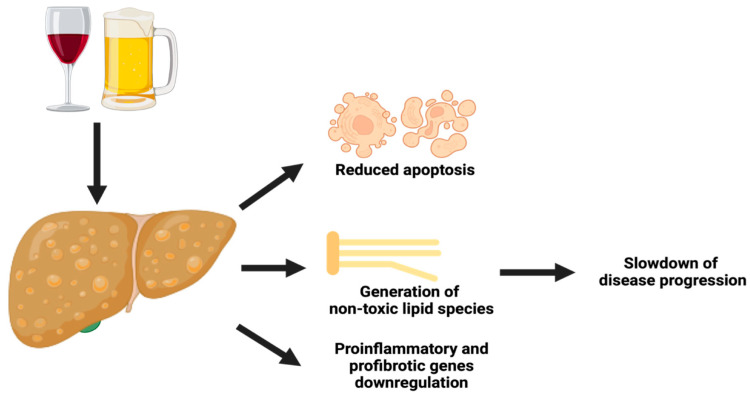
Biochemical pathways elicited by alcohol in NAFLD. Alcohol consumption increases the expression of anti-apoptotic genes and the generation of non-toxic lipid species (such as triglycerides enriched in monounsaturated fatty acids, with a decrease in palmitic acid, which exerts hepatotoxic effects). Conversely, the expression of pro-inflammatory and pro-fibrotic genes is reduced. This ultimately leads to a protective effect, slowing down NAFLD progression. Created with BioRender.com.

An experimental study on mice fed with a high-fat diet and ethanol-added water [48] suggested that alcohol induced the generation of non-toxic lipid species (including triglycerides enriched in mono-unsaturated fatty acids), reduced the expression of pro-inflammatory and pro-fibrotic genes, and restored mitochondrial function. A downregulation of several pro-apoptotic genes with a concomitant increased expression of anti-apoptotic genes was also detected. Regarding the histology, light alcohol consumption led to a reduced presence of fibrosis in portal areas. Fatty acid analysis revealed increased levels of triglycerides and phospholipids within larger lipid droplets, with a decrease in palmitic acid, known to be harmful to the hepatocytes (Figure 1).

## 4. Chronic Viral Hepatitis

Chronic hepatitis C virus (HCV) and hepatitis B virus (HBV) infections are a global health problem, affecting millions of people worldwide, with important morbidity and mortality [49,50,51,52,53].

HCV infection has a higher prevalence in patients with alcohol use disorder (AUD); in particular, a 30-fold higher prevalence has been estimated compared with the general population [54,55]. No similar evidence was found regarding the prevalence of HBV infection in alcohol users.

It has been widely demonstrated that alcohol abuse contributes to accelerate the progression of chronic HCV and HBV hepatitis [56,57], promoting worsening fibrosis, cirrhosis, and hepatocellular carcinoma [58].

The interactions between alcohol and hepatitis viruses in causing liver damage are complex, and the biological mechanisms involved have not yet been clearly defined [59].

Hepatitis B virus (HBV) infection leads to massive liver damage with progression, if untreated, to cirrhosis in 15–40% of patients, and consequently to liver failure and cancer [60]. 

Alcohol consumption accelerates liver injury in patients with chronic HBV infection, leading to an increase in fatty changes and fibrosis progression [61]. A study, performed in vivo [62], that analyzed the trend of fibrosis markers in immunologically compromised mice (SCID), thus in the absence of an immune response against viral proteins, fed an isocaloric diet with ethanol component for 5 weeks. An approximately 7-fold increase in HBV DNA load was found in this population compared with the control group. An increase in hepatitis B surface antigen (HBsAg) levels was also found. Mice with HBV fed with an alcohol-enriched diet showed significantly worsening of liver steatosis. Thus, the study found that chronic alcohol consumption alters, in vivo, gene expression and viral replication patterns, even in the absence of an immune response against the virus.

The mechanisms by which alcohol catalyzes liver damage in patients with chronic hepatitis B are still being investigated; however, several studies attempted to explain the molecular mechanisms underlying liver damage in these individuals.

**Figure 2:**
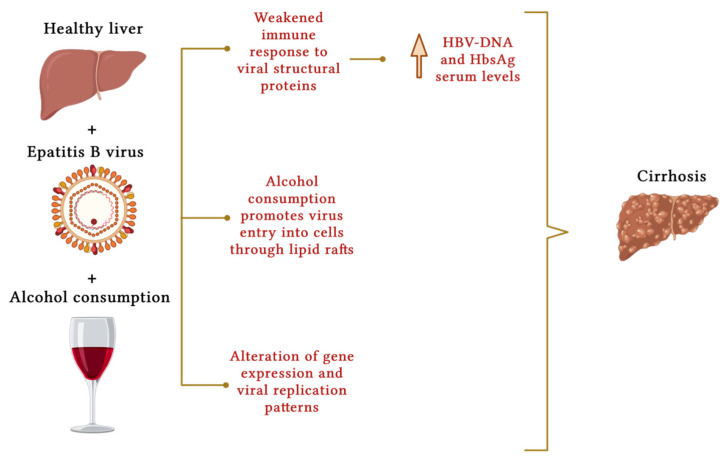
Molecular mechanisms that represent the contribution of alcohol consumption to the development of liver cirrhosis in chronic HBV infection. Alcohol consumption impairs the immune response to viral proteins and alters viral gene expression, promoting virus replication (with increased serum levels of HBV DNA and HBsAg). Alcohol consumption seems to promote viral entry into hepatocytes through lipid rafts. Created with BioRender.com.

Among the mechanisms investigated, interference with the immune system seems to impact the pathogenesis. Indeed, alcohol has been shown to impair a proper immune response to HBV, promoting its replication and liver inflammation. Specifically, studies have shown that excessive alcohol consumption can elevate HBV DNA serum levels by weakening the immune response to viral structural proteins [63] (Figure 2). In addition to the direct effect of alcohol on viral replication, it also appears to have an indirect role in the life cycle of the virus and its entry into cells via lipid rafts [64], which play an important role in molecular signaling mechanisms. Cell membranes have receptors that act as entry points for viruses, allowing them to enter hepatocytes. They are greatly affected by the surrounding microenvironment, which in turn is influenced by alcohol consumption, particularly by the production of acetaldehyde [65].

**Figure 3:**
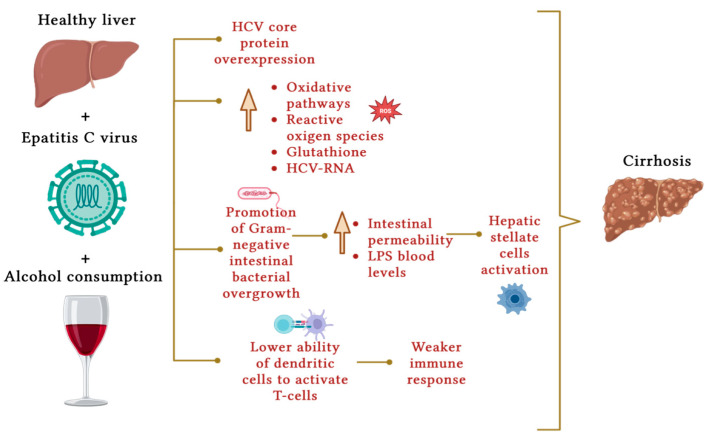
Molecular mechanisms that represent the contribution of alcohol consumption to the development of liver cirrhosis in chronic HCV infection. The overexpression of HCV core protein, the activation of oxidative pathways, and the depletion of molecules with antioxidant activity such as glutathione favoring the production of reactive oxygen species have been recognized as cofactors involved. Ethanol also alters intestinal permeability, leading to an increase in blood levels of lipopolysaccharide secondary to the growth of Gram-negative intestinal bacteria and liver fibrosis due to the activation of hepatic stellate cells. Finally, alcohol weakens the immune response as T cells are activated with greater difficulty by dendritic cells. Created with BioRender.com.

The pathophysiological mechanism by which alcohol consumption and hepatitis C infection synergistically accelerate the progression of liver fibrosis and liver damage also remains unclear [66]. Several hypotheses have been investigated (Figure 3). Otani et al. [67] believe that the combined detrimental effect resulting from the action of alcohol and chronic hepatitis C acts via HCV core protein overexpression, with the enhancement of oxidative pathways and reactive oxygen species. Chronic HCV infection has been shown to cause glutathione depletion and consequently oxidative stress [68].

Alcohol consumption promotes the growth of certain Gram-negative intestinal bacteria, increasing intestinal permeability and thus blood levels of lipopolysaccharide [69], activating hepatic stellate cells, which promote fibrosis [70]. Among the mechanisms involved, in patients with chronic HCV infection, dendritic cells have been shown to have a lower ability to activate T cells, consequently prompting a weaker immune response [71].

Some studies suggest that, in addition to the inhibitory effects on the immune system, alcohol consumption is also involved in the activation of inflammatory pathways and cytokines that mediate inflammation; this accelerates the progression of liver disease [72], demonstrating that even in individuals with chronic hepatitis C who consume moderate amounts of alcohol (<50 g/day), inflammatory markers are increased compared to controls, suggesting an important role of oxidative stress. 

The impact of modest alcohol consumption on increasing serum HCV RNA levels has also been demonstrated [73].

### 4.1. Effects of Alcohol Consumption on HBV-Related Liver Disease

There are multiple pieces of evidence of alcohol abuse-related prognostic worsening in patients with chronic hepatitis B (CHB). In a retrospective study conducted in Taiwan [74] that analyzed 966 cirrhotic patients with HBV infection, a significantly higher annual incidence of cirrhosis and HCC was documented in subjects with a history of concomitant severe alcohol consumption (>80 g/day for >5 years), compared with the control group. This finding is congruent with a prospective study [75] that analyzed 2352 HBsAg-positive patients for 20 years, documenting that alcohol abuse (>60 g/day) was associated with an approximately 6-fold increase in the risk of death from cirrhosis and HCC.

Regarding moderate alcohol consumption, the evidence is scant and shows a modest correlation. A cross-sectional study found an overlap in the incidence of liver fibrosis between those who do not consume alcohol and those who consume between 1 and 20 g/day [76].

A study conducted in China [77] did not document a significant effect of moderate alcohol consumption on liver cirrhosis in patients with hepatitis B. The study analyzed how different levels of alcohol consumption may impact liver cirrhosis in a population of 90 patients with chronic hepatitis B. Consumption was classified as absent, moderate (if less than 40 g/day for men and <20 g/day for women, and for less than 5 years), and excessive. At the time of enrollment and throughout the experimental period, patients stopped consuming alcohol; in addition, treatment with nucleoside analogs and antifibrotic agents (not otherwise specified) was introduced or continued. Indicators of the level of fibrosis (such as procollagen type III, collagen type IV, laminin, hyaluronic acid), in addition to the Child–Pugh score, number of complications, circulating HBV DNA and HBsAg, bilirubin, and transaminases were analyzed at 0, 3, and 6 months after quitting drinking and after pharmacological treatment. It was found that all indicators of fibrosis under investigation, especially serum HBV DNA, increased significantly in the group of patients with excessive alcohol consumption. In contrast, no difference was found between the groups with absent and moderate consumption. Transaminase levels in excessive alcohol users also returned to normal after treatment with nucleoside analogs and antifibrotic treatments.

A study conducted in North America and Canada [78] investigated how the adoption of certain lifestyle behaviors, such as alcohol, coffee, and tobacco consumption, can influence the clinical course of chronic hepatitis B. Clinical markers of the severity of liver damage, such as alanine aminotransferase (ALT) levels and Fibrosis-4 score (FIB-4), were analyzed in a group of 1330 subjects. Regarding alcohol consumption specifically, it was classified as absent, moderate (defined as consuming 12 or more drinks in the past year), and at risk. At baseline, 20.2% of participants were classified as moderate drinkers. In this cohort, in multivariate analysis after adjustment for potential confounders such as age, sex, or ethnicity, moderate alcohol consumption was not associated with markers of disease severity (ALT or FIB-4) or rates of worsening disease progression.

### 4.2. Effects of Alcohol Consumption on HCV-Related Liver Disease

There is strong evidence confirming that alcohol abuse worsens liver impairment [79]. Alcohol consumption > 50 g per day has been widely shown to be associated with the progression of liver fibrosis in patients with chronic hepatitis C [80,81].

In a retrospective study [82], patients with chronic hepatitis C were divided into two groups, based on whether their alcohol intake was significant (>40 g of alcohol/day in women and >60 g of alcohol/day in men for >5 years) or not significant; it was found that there was a two- to three-fold greater risk of liver cirrhosis and decompensated liver disease in the alcohol group. Also, the rate at which subjects developed cirrhosis was faster in the alcohol group, with 58% being cirrhotic by the second decade as opposed to 10% being cirrhotic in the non-alcohol group by the second decade.

For affected patients who consume alcohol moderately, there is not yet enough evidence to suggest that lower alcohol consumption worsens liver disease, although it appears that even minimal alcohol consumption (1–20 g/day) can accelerate the progression of liver disease. In fact, patients with chronic hepatitis C and alcoholic liver disease have more impaired biochemical function and a greater chance of developing cirrhosis.

Some cross-sectional studies have been conducted, including patients from France, Spain, and the United States. An association of histologically detectable fibrosis related to alcohol consumption < 50 g/day was found [83].

Discordant data emerged from a study [84] that analyzed a total of 857 patients with chronic hepatitis C by performing liver biopsies. This analysis showed that the main impact on the severity of liver fibrosis was due to the duration of HCV infection, whereas it was independent from moderate alcohol consumption (<30 g/day) when the duration of the infection was considered. Overlapping results were found between those who consumed alcohol moderately and those who never consumed alcohol. 

A study conducted on 349 patients with chronic hepatitis C in Northern Italy [85] revealed an increased risk of developing hepatic steatosis associated with HCV genotype 3a and alcohol consumption. Only patients who did not consume alcohol or had an intake of <40 g/day were included in the study, thus demonstrating that even moderate alcohol consumption can lead to liver damage. Another cross-sectional study [86] analyzed the correlation between the amount of alcohol intake and the evolution of liver fibrosis in 800 patients with chronic hepatitis C, quantifying current and past consumption. Alcohol consumption was classified as “light” if between 0 and 20 g/day, “moderate” between 20.1 and 50 g/day, and “abuse” if >50 g/day. Over 5 years of follow-up, statistically significant evidence of fibrosis progression emerged only in the case of alcohol abuse. However, a gradual increase in mean fibrosis and the odds ratio for fibrosis was also observed in patients consuming less than 50 g/day of alcohol.

A Swedish retrospective study [87] analyzed the progression of liver fibrosis in 78 patients with chronic hepatitis C who consumed low or moderate amounts of alcohol (median of 4.8 g/day). All patients underwent two liver biopsies with a median time between biopsies of 6.3 years; patients with a higher total amount of alcohol and drinking frequency showed worsening liver fibrosis over time, underlining the importance of the frequency rather than the amount of alcohol consumed. In patients who consumed alcohol in moderate or low amounts, occasional alcohol use appeared to be less harmful than daily use.

A cohort study conducted in South Korea [88] evaluated the correlation between alcohol consumption and mortality in both healthy patients and patients with chronic viral hepatitis. Data from 346361 subjects were analyzed based on screening evaluations.

Alcohol consumption was quantified as light (<10 g/day for women and <20 g/day for men), moderate (10–40 g/day for women and 20–60 g/day for men), and heavy (>40 g/day for women and >60 g/day for men). In patients with chronic viral hepatitis, the hazard ratios for all-cause mortality for light, moderate, and heavy alcohol consumers, respectively, were 1.19 (95% CI 1.05–1.36), 1.23 (95% CI 1.06–1.43), and 1.69 (95% CI 1.28–2.24). In this group, mortality was predominantly related to worsening liver disease.

In patients without chronic viral hepatitis, the hazard ratios were 0.92 (95% CI 0.87–0.98), 1.08 (95% CI 1.06–1.43), and 1.51 (95% CI 1.33–1.72), respectively. Therefore, even light-to-moderate alcohol consumption in patients with chronic hepatitis C appears to be burdened by increased all-cause mortality.

In contrast to this meager evidence, moderate alcohol consumption is associated with cardiovascular risk reduction, based on the beneficial effects on lipid control and thrombosis.

A survey conducted in 2013 [89] analyzed the impact that moderate alcohol consumption in patients with chronic hepatitis C may have on all-cause mortality, liver disease, and cardiovascular disease. Specifically, the number of drinks consumed in the previous 12 months and the number of days per month on which the patient reported consumption were analyzed. Alcohol consumption was defined as moderate if between 1 and 19 g/day, excessive if >20 g/day, and heavy if >30 g/day. The cohort under study included 8985 patients, of whom 218 had chronic hepatitis C. Patients with chronic hepatitis C showed increased mortality, both related to liver disease and all causes; in particular, a mortality rate of 19.9% at 162.95 months was reported in patients with chronic hepatitis C compared with 11.37% at 175.49 months in controls, which was even higher in the case of moderate (1–19 g/day) or heavy alcohol intake (>30 g/day).

Specifically, in the multivariate Cox proportional hazards model, in patients with moderate alcohol consumption, both the overall risk of death (HR 2.44) and liver-related mortality (HR 74.25) were increased, and no statistically significant association was found with cardiovascular mortality (HR 0.71); in these patients, moderate consumption did not seem to have a potential cardioprotective effect, and the risk of liver disease overcame any potential cardiovascular benefit. In patients with excessive alcohol consumption, both the risk of overall mortality (HR 5.12), liver-related mortality (HR 183.74), and cardiovascular-related mortality (HR 3.34) were markedly increased.

Therefore, a safe level of alcohol consumption in patients with chronic hepatitis C could not be documented. Even mild or moderate consumption appears to be associated with the progression of fibrosis and its complications.

Finally, it seems that prior alcohol consumption is not a predictor of HCV treatment failure, and active alcohol consumption appears to be related to treatment discontinuation more than to treatment success, regardless of the degree of alcohol intake [90]. Another recent, less extensive report [91] found a lower response to therapy with standard interferons in subjects who had previously consumed more than 30 g of alcohol per day; in contrast, no reduction in response to direct-acting antiviral (DAA) therapy was found in those who consumed alcohol [92].

### 4.3. HIV Coinfection

Many studies have shown that patients co-infected with HCV and HIV have faster progression to fibrosis [93], even before the decline of CD4 count. It has been reported that an HIV-infected patient with less than 200 CD4/mL and consuming more than 50 g of alcohol daily has a median expected time to development of cirrhosis of 16 years, unlike HIV-infected patients with more than 200 CD4/mL and consuming 50 g or less of alcohol daily, who have a median time to progression of 36 years [94].

An observational study [95] analyzed the risk of liver fibrosis progression with different levels of alcohol consumption in patients with HCV–HIV coinfection, HIV mono-infection, HCV mono-infection, or uninfected. Alcohol consumption has been classified as non-hazardous, hazardous/binge drinking (greater than or equal to 6 drinks on a single occasion), or abuse.

Non-invasive markers were used to analyze the degree of liver fibrosis, specifically using the Fib4 index. The study showed a more rapid progression of fibrosis as alcohol consumption increased, with a decreasing dose response among HIV/HCV-coinfected, HIV mono-infected, HCV mono-infected, and uninfected subjects.

A decrease in CD4 count and an increase in HCV RNA serum levels were found as alcohol consumption increased.

In contrast with previous findings, it has been reported in women with HIV–HCV coinfection that light-to-moderate alcohol consumption (<7 drinks/week) was not associated with substantial worsening of liver fibrosis measured by Fib4 compared with abstinent subjects. In contrast, fibrosis progression was documented in cases of alcohol abuse (>7 drinks per week) [96].

## 5. Hemochromatosis

A toxic additive effect has been hypothesized in subjects with hemochromatosis in the case of concurrent alcohol consumption, partly because iron accumulation in the liver has often been observed in alcoholics, with a mechanism not yet fully understood [97,98].

Fletcher et al. estimated an approximately 6-fold increased risk of developing liver cirrhosis in individuals with hemochromatosis consuming more than 60 g/day of alcohol [99].

An in vivo study, which produced conflicting results, was conducted on 59 mice [100] and reported a lower bioavailability of vitamins (especially vitamin E) in all animals exposed to an iron-containing diet and alcohol consumption (at doses of 2%, 5%, and 8%). A significant increase in lipid peroxidation activity was found in all animals consuming alcohol, an iron-rich diet, or both. Blood levels of transaminases showed a greater increase in the case of an iron diet alone when compared to mice fed with ethanol and an iron diet.

The study found that ethanol exposure in iron-overloaded animals positively modulated oxidative stress and organ damage, and this finding was also confirmed by liver biopsy. Notably, in animals fed an iron-rich diet with ethanol in addition, the reduction in vitamin C levels appeared to be much less pronounced when compared to the group only fed an iron-rich diet, which would suggest an antioxidant effect of ethanol in the case of iron overload, despite the reduction in antioxidant vitamins.

Studies on moderate alcohol consumption in hemochromatosis are therefore still lacking.

## 6. Conclusions

The extremely heterogeneous results from the available studies make it difficult to obtain a clear indication of the effects of modest drinking. The definition of minimal alcohol consumption itself constitutes a non-negligible problem, as demonstrated by the different thresholds used by researchers. Considering the mortality of patients with NAFLD, only a few authors concluded that low alcohol intake could reduce death by all causes without an increase in adverse liver outcomes. These protective effects seem to be more evident in wine drinkers, and they might be mediated by a beneficial effect on the cardiovascular system. Concordant data emerged from many studies in relation to carcinogenesis, which is favored even by small amounts of alcohol. Univocal evidence about the deleterious consequences of binge drinking resulted from several works.

Although numerous studies documented the extent of liver damage catalyzed by alcohol abuse in individuals with chronic HBV or HCV infection, there is currently limited evidence on the consequences of moderate consumption in this particular group of patients. Studies on this topic, conducted by analyzing direct or indirect markers of organ function in viral-infected subjects who consume moderate amounts of alcohol, are numerically few with meager evidence, and they also have major biases, which may compromise the applicability of the outcomes. The majority of these works, however, agrees on the identification of alcohol consumption as a factor responsible for more abrupt disease progression in a dose-dependent manner. The biological and molecular mechanisms underlying alcohol consumption-related liver damage in the case of patients with chronic HBV and HCV infection are still being analyzed and include suppression of the immune response, oxidative stress, stimulation of intestinal bacterial overgrowth, and activation of inflammatory pathways. Thus, it seems that there is no safe level of alcohol consumption in this class of patients because even moderate consumption seems to be related to the activation of biomolecular mechanisms that can trigger the progression of liver damage.

An additive toxic effect of alcohol in hemochromatosis has been supposed. However, few studies have been conducted in this regard, so the evidence is still lacking. Further studies with a more rigorous methodological approach are indispensable in order to clarify the real impact of low alcohol consumption in this setting.

## Figures and Tables

**Table 2:** Histological-based studies.

Reference	Population Sample	Modest Alcohol Use Definition	Main Results
Ajmera et al. 2018 [35]	285	<30 g/d in men <20 g/d in women	modest drinkers have less NASH resolution and less improvement in steatosis
Dunn et al. 2012 [36]	583	<30 g/d in men <20 g/d in women	protective effect against the development of NASH
Kimura et al. 2018 [37]	301	<20 g/d	higher prevalence of cirrhosis and incidence of HCC in mild drinkers
Ascha et al. 2010 [38]	195	<2 drinks/d or 3–6 drinks on weekend	alcohol use is a risk factor for the onset of HCC
Dixon et al. 2001 [39]	105	<200 g/w	protective effect against the development of NASH
Cotrim et al. 2009 [40]	132	<40 g/d	no beneficial effect
Mitchell et al. 2018 [41]	187	<70 g/w	modest alcohol intake is a protective factor against advanced fibrosis
Ekstedt et al. 2009 [42]	71	<30 g/d in men <20 g/d in women	heavy episodic drinking causes fibrosis progression
Kwon et al. 2014 [43]	77	<40 g/years	protective effect against fibrosis in drinkers of more than 10 g/y (up to 40 g/y)

## Data Availability

Not applicable.
